# Finite-element analysis-based design and efficacy assessment of a three-dimensional anisotropic heel cushioning pad for diabetic foot management

**DOI:** 10.3389/fbioe.2025.1694935

**Published:** 2025-11-19

**Authors:** Xiong-Gang Yang, Xing-Xi Hu, Lang-Tao Ma, Ming-Qi Jiang, Gui-Qian Zhang, Sheng Lu

**Affiliations:** 1 Department of Orthopedics, the First People’s Hospital of Yunnan Province, the Affiliated Hospital of Kunming University of Science and Technology, Kunming, Yunnan, China; 2 The Key Laboratory of Digital Orthopedics of Yunnan Province, Kunming, Yunnan, China; 3 Intelligent Orthopedic Medical Technology Research Center, Kunming University of Science and Technology, Kunming, Yunnan, China; 4 Department of Orthopedic and Trauma Surgery, The Affiliated Hospital of Yunnan University, Kunming, China

**Keywords:** diabetic foot, finite-element analysis, heel cushion pad, anisotropy, elasticity modulus

## Abstract

**Background:**

Diabetic foot ulcer (DFU) is a common complication observed in diabetic patients, which can lead to lower limb amputation in severe cases and seriously impair the patient’s mobility and even endanger life. Plantar insoles aim to redistribute pressure, yet diabetic foot tissues exhibit altered material properties, necessitating a novel approach to address vertical pressures and shear forces. This study sought to design a three-dimensional anisotropic heel cushioning pad that mitigates both vertical pressure and anteroposterior/mediolateral (AP/ML) shear forces.

**Methods:**

CT data of the foot were collected and stored in DICOM format. We reconstructed a foot model and simulated the heel cushioning pad with varying elastic moduli in compressive, AP-shear, and ML-shear directions. We used finite-element analysis (FEA) to assess the impact of these moduli on peak stresses under various loading conditions. The data were fitted with a polynomial, and a regression equation was obtained.

**Results:**

Reducing the elastic moduli of heel cushioning pads led to decreased peak stresses across all directions. Notably, the peak compressive stress decreased by 52.20%–66.91%, while AP and ML shear stresses decreased by 51.05%–75.58% and 54.16%–72.42%, respectively. Polynomial analyses revealed optimal stress reductions within specific elastic modulus ranges (400, 800, and 1,000 kPa in compressive, AP-shear, and ML-shear dimensions, respectively), indicating diminishing benefits beyond these points.

**Conclusion:**

FEA revealed that heel cushioning pads with tailored elastic moduli can significantly reduce peak stresses, with diminishing benefits observed beyond certain thresholds. These findings suggest that selecting materials with elastic moduli just before the plateau could yield the most effective cushioning for DFU prevention, offering valuable insights for industrial applications.

## Introduction

Diabetes mellitus (DM) has rapidly evolved into a global epidemic disease, affecting nearly 10.5% of the adult population worldwide (International Diabetes Federation, 2021). With continuous improvements in living standards and the aging of the population in China, the number of diabetic patients is steadily increasing, currently reaching 130 million on the Chinese mainland, accounting for approximately one-third of the total number of cases globally (Li et al., 2020). Diabetes is associated with the development of complications throughout multiple systems of the body, among which diabetic foot ulcer (DFU) is one of the most common complications. The lifetime risk of developing DFU in diabetic patients is as high as 15%–25% (Chiwanga and Njelekela, 2015). The pathogenesis of DFU includes peripheral neuropathy, hyperglycemia, microvascular damage, reduced angiogenesis, alterations in the biomechanics of plantar tissues, and abnormal gait, leading to increased plantar pressure and shear forces (Bowering, 2001). Currently, the primary treatments for DFU include debridement, wound dressing, infection control, and offloading of the foot. However, the treatment outcomes for DFU are often unsatisfactory, with many patients ultimately requiring lower-limb amputation within 4 years of diagnosis (Singh et al., 2005). The increasing incidence of DFU and the related costs of treatment, nursing, and rehabilitation have placed a heavy burden on medical insurance and society. It is reported that up to one-third of the medical expenses in diabetes care are directly related to the treatment of DFU (Armstrong et al., 2020). Numerous guidelines and studies clearly indicate that DFU is a preventable complication, and early multidisciplinary care can prevent amputation in nearly half of the affected patients (Bus et al., 2020a).

Current assistive devices for the prevention or treatment of DFU primarily include the following categories: (1) total contact casting (TCC): it disperses and transfers plantar pressure to the entire lower extremity, offering significant plantar pressure cushioning, and is recognized as the “gold standard” for treating DFU ulcers (Sibbald and Ayello, 2019; Bhatt et al., 2021; Vierhout et al., 2022). However, it cannot be adjusted during use and often needs replacement, greatly affecting the patient’s daily activities; (2) removable casts/braces: they perform similar functions to TCC but have the advantages of being removable, being more tolerable, and having higher compliance (Piaggesi et al., 2016; Faglia et al., 2010; Bus et al., 2018); (3) therapeutic shoes/boots: they redistribute plantar pressure/shear force and reduce peak pressure, pressure–time integral, and shear force in high-risk areas (López-Moral et al., 2019; Preece et al., 2017; Chantelau et al., 1993); (4) therapeutic insoles: they are currently a research hotspot, with the advantages of being lightweight, highly compliant, cost-effective, and capable of redistributing plantar pressure and cushion the plantar shear stress, thus reducing the risk of ulcer formation (Jafarzadeh et al., 2021; Ahmed et al., 2020; Collings et al., 2020; Chanda and Unnikrishnan, 2018; Viswanathan et al., 2004; Lavery et al., 2012). The International Working Group on the Diabetic Foot (IWGDF) guidelines recommend the use of cushioning devices for pressure relief to prevent the formation of plantar calluses in all individuals at risk of developing DFUs (IWGDF-1∼3 grades), such as shoes, insoles, or toe orthoses (Bus et al., 2020b). A large number of biomechanical studies have clearly demonstrated a strong correlation between the occurrence of DFU and plantar pressure. Therefore, as recommended by the IWGDF guidelines, a substantial number of recent research studies have emerged, considering plantar offloading as a key factor in preventing the occurrence and recurrence of DFU and striving to explore various therapeutic tools such as ankle–foot orthoses, shoes, and insoles (Jafarzadeh et al., 2021; Ahmed et al., 2020; Collings et al., 2020; Chanda and Unnikrishnan, 2018; Viswanathan et al., 2004; Lavery et al., 2012).

In the design of pressure-relieving insoles, researchers primarily focus on the following aspects: (1) geometric shape and structure of the insoles: good conformity between the insoles and the plantar surface can achieve a uniform distribution of plantar pressure; (2) selection of insole materials: commonly used materials include silicone, Poron^®^ foam, EVA, Plastazote^®^ foam, PPT plastic, and Pelite^®^ foam; (3) stiffness and hardness of the material: these properties significantly affect the buffering effect of the insert. There are many types of insoles available for plantar pressure relief, but due to the technical difficulty of implementation and insufficient attention to shear force in previous studies, insoles specifically designed for shear force buffering are less commonly found in literature reports and available products.

It was indicated that only 38% of DFU patients have plantar ulcer locations that match peak pressure areas, suggesting that elevated peak pressure is not the sole factor leading to DFU (Veves et al., 1992). Furthermore, 41% of callus patients who wear therapeutic plantar pressure-relieving footwear in their daily lives still experience recurrent calluses (Scirè et al., 2009). Thus, simple plantar pressure relief may not achieve the most ideal DFU prevention effect for most patients. In our preliminary research, based on synchronous monitoring of 3D digital image correlation technology and plantar three-dimensional force plates, we tested the three-dimensional material properties of the heel soft tissue in diabetic patients and found that not only did they differ significantly from healthy young people in terms of material properties in the vertical compression direction, but significant changes were also observed in the anteroposterior (AP) and mediolateral (ML) shear directions. Therefore, to prevent development of DFU in diabetic patients, in addition to achieving ideal pressure relief in the vertical compression direction, providing effective shear force buffering in the AP and ML directions and restoring plantar mechanical conditions as close as possible to those of healthy individuals can bring additional benefits to patients. At the same time, we found that the material properties of plantar soft tissue in the vertical, AP, and ML dimensions are not isotropic but exhibit extremely significant differences. This suggests that the approach of treating therapeutic insoles as three-dimensional isotropic entities solely from the perspective of normal compression in previous studies is not entirely ideal. So far, no researchers have reported designing shoe inserts that provide plantar buffering effects in three dimensions based on the anisotropic material properties of plantar soft tissue.

Therefore, the primary aim of this research was to create novel anisotropic heel cushion pad designs to enhance both pressure and shear force buffering capabilities. Through finite-element analysis (FEA), we would investigate the impact of gradient elastic moduli on these buffering effects, ultimately guiding the selection of materials for industrial production of diabetic foot cushioning insoles.

## Materials and methods

### Finite-element (FE) model building

One case was enrolled after excluding diseases of bone and joint deformity, abnormal arch, fracture, tumor, or infection and a prior history of trauma or surgery. A 64-slice spiral CT scanner was used to conduct a CT scan of the foot. The scanning parameters were set as follows: layer thickness (1 mm) and resolution (0.25 mm), with the foot positioned in a neutral stance. The scanned data were stored in DICOM format files. The participant was fully informed about the study content, and the experimental procedures received approval from the Institutional Ethics Committee.

**FIGURE 1:**
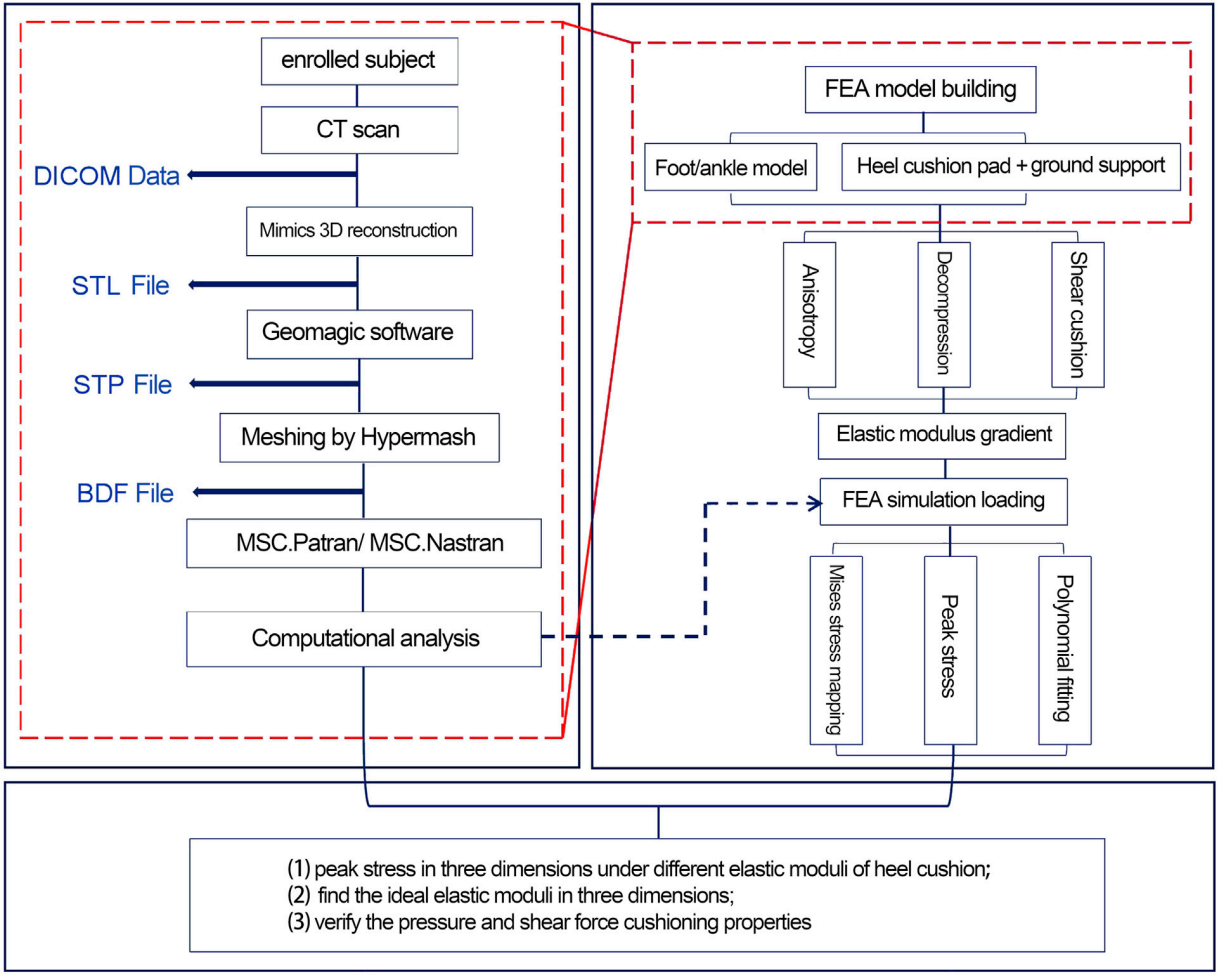
Flowchart for the design and verification of the anisotropic heel cushion pad for diabetic feet via finite-element analysis (FEA).

A flowchart illustrating the design of the anisotropic heel cushion pad for diabetic feet using FEA is shown in Figure 1. Using Mimics 21.0 software (Materialise Company, Belgium), the collected DICOM data were extracted, and a three-dimensional reconstruction of the bones, soft tissues, and skin was performed to obtain an STL structural model. Subsequently, the model was imported into Geomagic Studio 2014 software (Raindrop Company, America), where surface patching, noise reduction, and model smoothing were completed. Through reverse engineering, an STP-format geometric solid model of the foot was obtained. Hypermesh 14.0 software (Altair Company, America) was then used to import the STP file and complete mesh division, saving the file in the BDF format for subsequent use in FE pre-processing software MSC.Patran 2019 (NASA Company, America) and post-processing software MSC.Nastran 2019 (NASA Company, America). These programs were used to set mesh properties, define material parameters, apply mechanical loads, constrain boundary conditions, and conduct load analyses, for corresponding working conditions.

**FIGURE 2:**
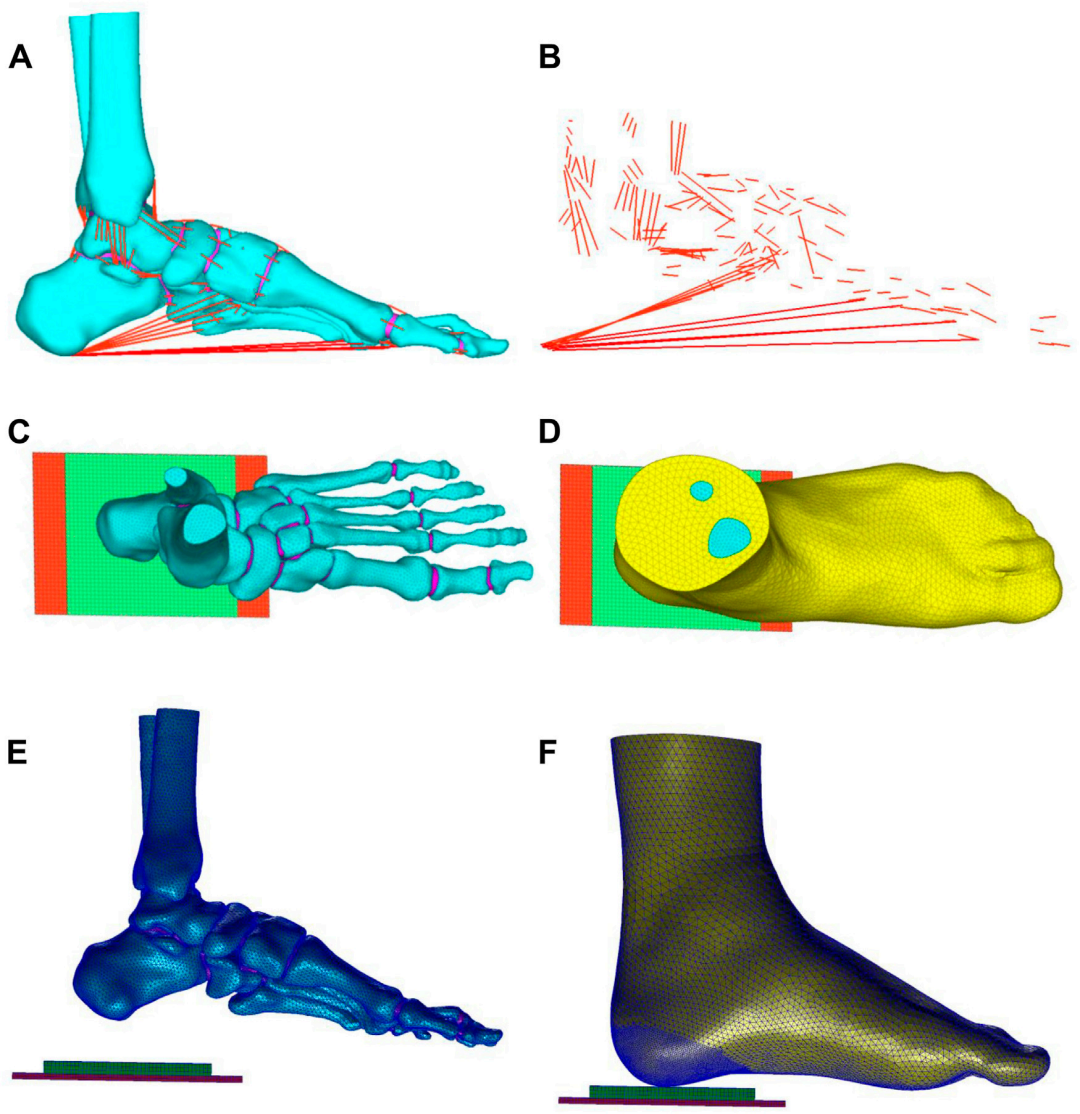
**(A,B)** Ligaments and tendons of the foot (red lines), connecting nodes on the bone grid surface and attachment points of ligaments and tendons, corresponding to MRI data to form 1D spring units as the tension elements of ligaments and tendons. Top view **(C,D)** and side view **(E,F)** of the three-dimensional finite element model: the model comprises bones (cyan part), skin and soft tissue (yellow part), tendons and ligaments (red lines), ground support layer (red block), and heel cushion pad (green block).

Figure 2 illustrates the schematic representation of the three-dimensional FE model developed in this study, comprising a total of 81,885 nodes and 388,128 elements. The FE model encompasses the following components: (1) skin and soft tissue; (2) skeletal system, including tibia, fibula, talus, calcaneus, navicular, cuboid, medial/intermediate/lateral cuneiforms, metatarsals, and phalanges; (3) articular cartilages; and (4) ligaments and tendons, which were configured as 1D linear spring elements in pre-processing software MSC.Patran 2019. Additionally, two rectangular parallelepiped thin structures were modeled to simulate the heel cushion pad (85 mm × 60 mm × 6 mm) and the ground support plate (150 mm × 75 mm × 3 mm). The bones, articular cartilage, skin, and soft tissue were meshed using TetMesh Tet4 Element tetrahedral solid elements. The element size of bones was set as 1.0–2.30 mm. The average element size of skin and soft tissue was 0.5–3.5 mm, with enhanced grid density (gradually refined from 2.0 mm to 0.5 mm, and the stress variation range is confirmed to be less than 1%, meeting the grid convergence requirements of the FE calculation) at the critical load-bearing heel region to optimize contact and friction mechanics. The ground support plate and heel cushion pad were meshed using Hex8 Element hexahedral solid elements, with a mesh size of 2 mm. The FE model has undergone mesh convergence analysis. All mesh elements have no quality issues such as deformity or abnormal aspect ratio (warpage rate <10°, aspect ratio ≤5, minimum acute Angle ≥30°, Jacobian coefficient >0.7, and taper >0.5).

### Material parameters and boundary condition setup

**FIGURE 3:**
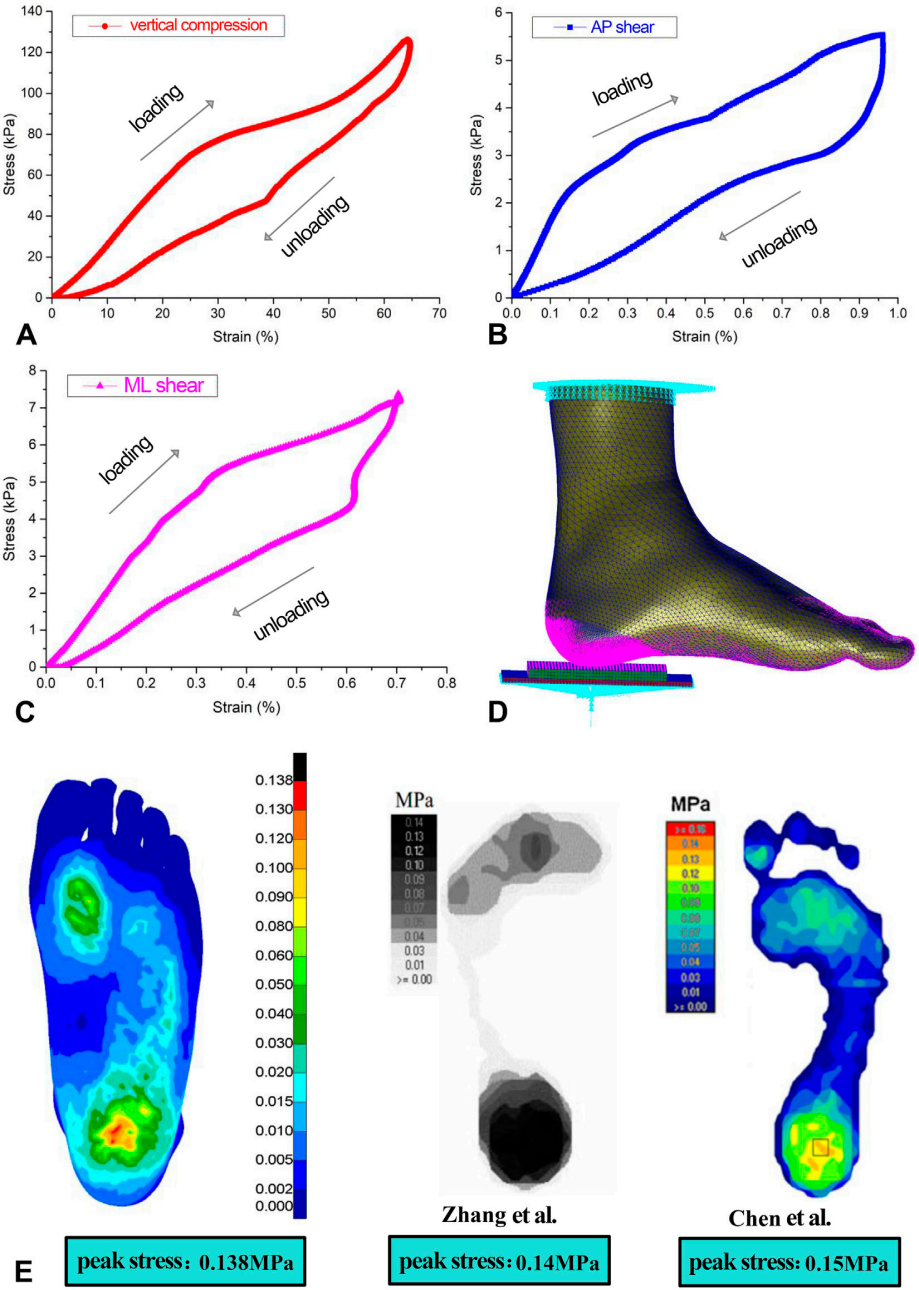
**(A–C)** Stress–strain constitutive relationship curve of the three-dimensional material properties of plantar soft tissue under the heel, including loading and unloading curves. **(D)** Boundary condition settings of the finite-element model. **(E)** Validation of the accuracy of the finite-element model, showing similar trends in plantar pressure distribution as two previous studies, indicating that the model is accurate and reliable. AP, anterior–posterior; ML, mediolateral.

The material parameter settings for the FE model were based on previously published literature (Jafarzadeh et al., 2021; Chen et al., 2015; Shaulian et al., 2021; Tang et al., 2019), assuming that the bones, articular cartilages, ground support plate, and heel cushion pad were isotropic, homogeneous, and continuous linear elastic materials, while the soft tissue under heel was a hyperelastic material. The material parameters for each non-tendon/ligament component of the FE model are detailed in Table 1. The material properties of the plantar soft tissue were set according to the stress–strain constitutive curves measured in three dimensions for diabetic patients in previous research (as shown in Figures 3A–C and Supplementary null Appendix 1). The material properties of heel cushion pads were preset with a series of gradient elastic moduli in three dimensions. The ligaments and tendons were modeled as 1D linear spring elements, with their elastic moduli and spring equivalent stiffnesses detailed in Supplementary Table S1. The bonded contact was set between the bones and articular cartilage, between the soft tissue and articular cartilage/bones, and between the heel cushion pad and ground support plate. The frictional contact with a coefficient of 0.5 was set between the heel cushion pad and the plantar soft tissue. The upper tibial and fibular cross-section was fixed, restricting six degrees of freedom to immobilize the proximal end of the model. The boundary constraints of the FE model are illustrated in Figure 3D.

**TABLE 1:** Material parameters of each component of the finite-element model.

Component	Elastic modulus (MPa)	Poisson’s ratio
Bones	7,300	0.30
Soft tissue under heel	Set according to the stress–strain constitutive curves measured in previous research for diabetic patients in three dimensions (Figures 3A–C and Supplementary null Appendix 1)	
Articular cartilages	1.01	0.40
Heel cushion pad	Gradient elastic moduli were set in three dimensions: (1) compressive: 1,000, 800, 600, 400, and 200 kPa; (2) AP shear: 1,500, 1,250, 1,000, 750, and 500 kPa; (3) ML shear: 2000, 1700, 1,400, 1,100, and 800 kPa	0.45
Ground support pad	17,000	0.10

Following the methodologies of Zhang et al. (2007) and Chen et al. (2015), the same boundary conditions were applied in this study, resulting in the distribution of plantar soft tissue compressive stress (as shown in Figure 3E) and a peak compressive stress value. Areas of high stress were concentrated around the heel region and the forefoot regions, with the highest stress located in the heel area. The maximum compressive stress peak (0.138 MPa) was similar to that in the findings of Zhang et al. (2007) (0.14 MPa) and Chen et al. (2015) (0.15 MPa), indicating an effective and reliable model (as shown in Figure 3E).

### Computer simulation loading test

A series of gradient elastic modulus values were predefined for the heel cushion pad across three dimensions (see Table 1), and different ground reaction forces were applied to the bottom of the heel in a three-dimensional manner through the ground support board. These forces were set based on the peak force experienced by the heel soft tissue during the stance phase of walking, with vertical pressure, AP shear force, and ML shear force set at 300 N, 25 N, and 15 N, respectively. This study used static simulations based on quasi-static gait loading conditions. Mises stress mappings were drawn, and the peak compression and AP/ML shear stresses on the plantar surface of heel were observed. When applying vertical compressive force, no lateral shear force was loaded, whereas when applying AP and ML shear forces, a 300 N vertical compressive force was applied simultaneously.

### Data processing and statistical analysis

Line graphs depicting the relationship between the elastic moduli of heel cushion pads and the peak stresses on plantar surfaces were constructed. The data were subjected to polynomial fitting using the “curve fitting” module in MATLAB R2021a software (MathWorks, Massachusetts, USA), yielding a regression equation to continuously predict the impact of the elastic moduli of the heel cushion pad on the peak stress on the plantar surface. Residual plots were also generated, and the goodness of fit of the model was assessed using the coefficient of determination R^2^ and the root-mean-square error (RMSE). The closer R^2^ is to 1 or RMSE to 0, the higher the goodness of fit of the model, indicating greater reliability of the model.

## Results

### FEA in the vertical compression direction

**FIGURE 4:**
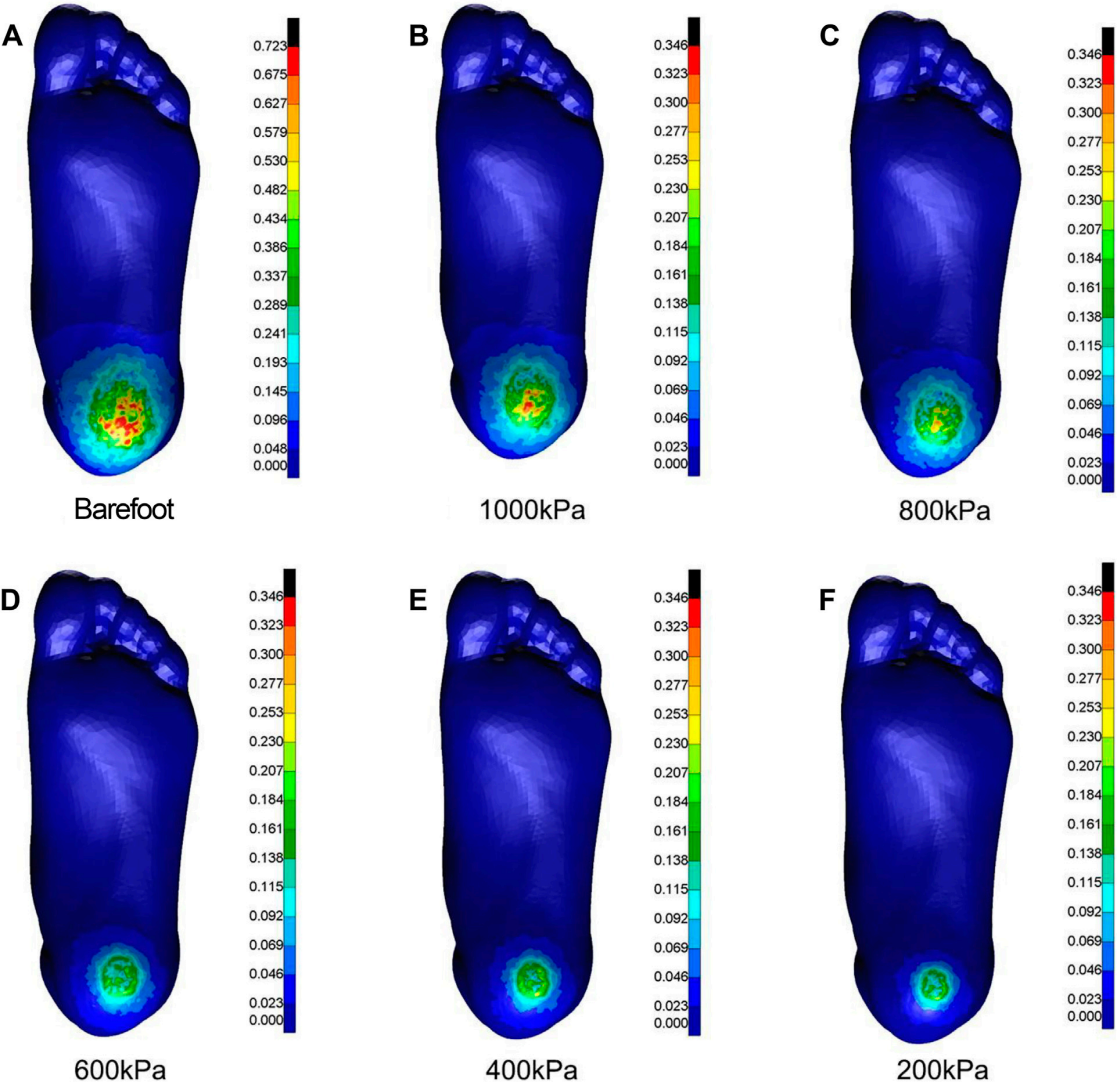
Cloud maps of Mises stress under vertical compression towards different heel cushion elastic moduli (**B**: 1000kPa; **C**: 800kPa; **D**: 600kPa; **E**: 400kPa; **F**: 200kPa) compared to barefoot state **(A)**. The color bar unit is MPa.

**FIGURE 5:**
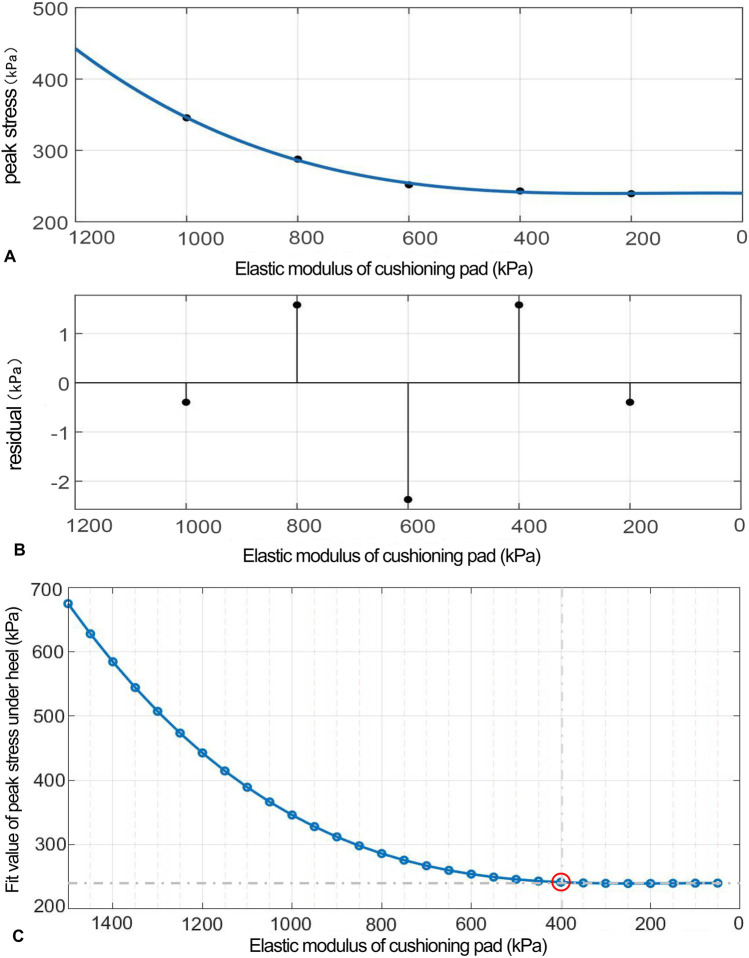
**(A)** Polynomial fitting curve between the peak compressive stress and elastic modulus of the heel cushion pad. **(B)** Residual plot. **(C)** Using the fit equation, the change curve of predicted peak compressive stress on the plantar surface with changes in the vertical elastic modulus of the heel cushion pad within the range of 50–1,500 kPa. When the elastic modulus decreases to approximately 400 kPa, the peak stress of the plantar surface gradually reaches a plateau.

As the elastic modulus of the heel cushion pad decreased, the distribution of compressive stress in the stress cloud image also reduced (see Figure 4). Compared to barefoot conditions, the heel cushion pad with a compressive elastic modulus of 200–1,000 kPa demonstrated a significant pressure-buffering effect, with a notable reduction in peak compressive stress (see Supplementary Figure S1A; Table 2, decreasing from 723.15 kPa to a range of 345.68–239.31 kPa, representing a decrease of 52.20%–66.91%). Moreover, as the elastic modulus of the heel cushion pad uniformly decreased, the rate of decline in plantar peak compressive stress gradually slowed, indicating that the relationship between the two is not simply linear. Therefore, polynomial fitting was performed to illustrate the relationship between the two, as shown in Figure 5A, resulting in the following regression equation:

$$f\left(x\right)=p_{1}*x^{3}+p_{2}*x^{2}+p_{3}*x+p_{4}.$$

**TABLE 2:** Relationship between the peak compressive stress and elastic moduli of the heel cushion pad.

Elastic modulus (kPa)	Peak compressive stress (kPa)	Percentage decline (compared to barefoot)
Barefoot	723.15	0.00%
1,000	345.68	52.20%
800	287.74	60.21%
600	251.82	65.18%
400	243.16	66.37%
200	239.31	66.91%

(“x” is standardized using a mean of 600 and a standard deviation of 316.2. The regression coefficients and their respective 95% confidence intervals [95% CIs] are as follows: p1 = 5.669 [−38.14, 49.47], p2 = 24.19 [−3.912, 52.28], p3 = 32.98 [−30.2, 96.15], and p4 = 254.2 [224.9, 283.5]).

The fitted regression line closely matches the original data points, as indicated by the small residuals in the residual plot (Figure 5B), suggesting excellent model fitting. The adjusted R^2^ value is 0.9945, and the RMSE is 3.31, further confirming the excellent goodness of fit. Using the above regression equation, a curve graph is drawn to predict the plantar peak compression stress as the elastic modulus varies within the interval of 50–1,500 kPa (Figure 5C). The graph shows that as the elastic modulus decreases, the reduction trend in plantar peak stress slows down. When the elastic modulus decreases to approximately 400 kPa, the plantar peak stress begins to reach a plateau, approaching a horizontal asymptote, with a predicted peak stress value of 241.58 kPa, reflecting a 66.59% decrease compared to barefoot conditions.

### FEA in AP shear direction

**FIGURE 6:**
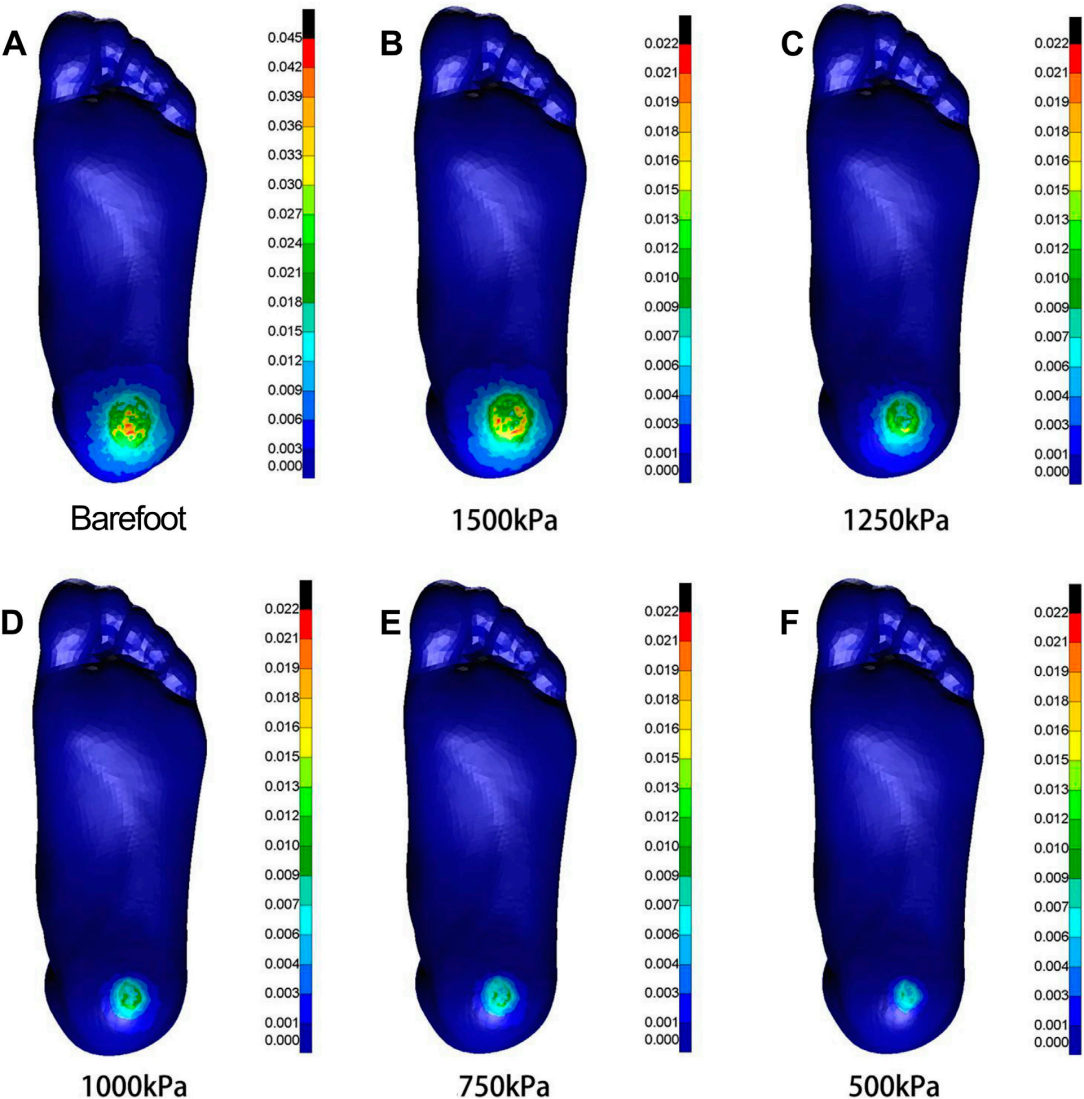
Cloud maps of Mises stress under anterior-posterior shear towards different heel cushion elastic moduli (**B**: 1500kPa; **C**: 1250kPa; **D**: 1000kPa; **E**: 750kPa; **F**: 500kPa) compared to barefoot state **(A)**. The color bar unit is MPa.

**FIGURE 7:**
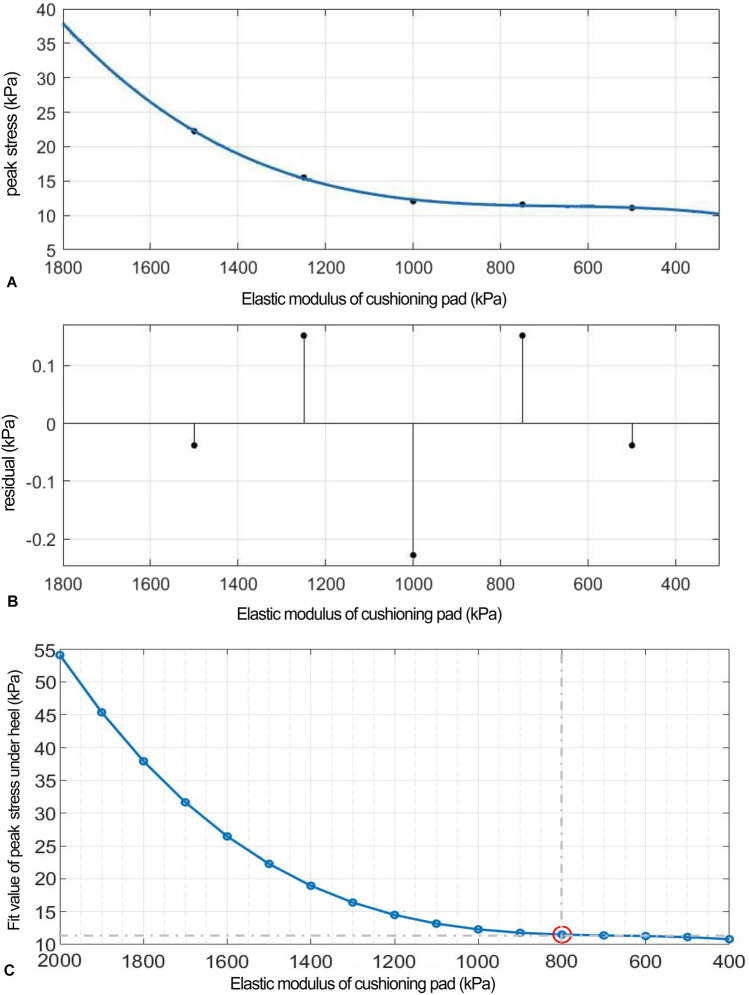
**(A)** Polynomial fitting curve between peak shear stress and shear elastic modulus of the heel cushion pad. **(B)** Residual plot. **(C)** Using the fit equation, the change curve of predicted peak shear stress on the plantar surface with changes in the anterior–posterior shear elastic modulus of the heel cushion pad within the range of 400–2000 kPa. When the elastic modulus decreases to approximately 800 kPa, the plantar peak shear stress gradually reaches a plateau. AP, anteroposterior.

As the elastic modulus of the heel cushion pad decreased, the distribution of AP shear stress in the stress cloud image also reduced (see Figure 6). Compared to barefoot conditions, the heel cushion pad with an AP shear elastic modulus of 500–1,500 kPa exhibited a significant AP shear buffering effect, with a notable reduction in peak shear stress (see Supplementary Figure S1B; Table 3, decreasing from 45.41 kPa to a range of 11.09–22.23 kPa, representing a decrease of 51.05%–75.58%). Additionally, as the elastic modulus of the heel cushion pad uniformly decreased, the rate of decline in plantar peak stress gradually slowed, indicating that the relationship between the two is not simply linear. Therefore, a polynomial fitting was performed to illustrate the relationship between the two, as shown in Figure 7A, resulting in the following regression equation:

$$f\left(x\right)=p1*x^{3}+p2*x^{2}+p3*x+p4.$$

**TABLE 3:** Relationship between the peak AP shear stress and elastic moduli of the heel cushion pad.

Elastic modulus (kPa)	Peak shear stress (kPa)	Percentage decline (compared to barefoot)
Barefoot	45.41	0.00%
1,500	22.23	51.05%
1,250	15.52	65.82%
1,000	12.07	73.42%
750	11.58	74.50%
500	11.09	75.58%

Footnote: AP, anteroposterior.

(“x” is standardized using a mean of 1,000 and a standard deviation of 395.3. The regression coefficients and their respective 95% CIs are as follows: p1 = 1.074 [−3.134, 5.282], p2 = 2.75 [0.05, 5.45], p3 = 2.69 [−3.38, 8.75], and p4 = 12.3 [9.48, 15.11]).

The fitted regression line closely matches the original data points, as indicated by the small residuals in the residual plot (Figure 7B), suggesting excellent model fitting. The adjusted R^2^ value is 0.9953, and the RMSE is 0.318, further confirming the excellent goodness of fit. Using the above regression equation, a curve graph is drawn to predict the plantar AP peak shear stress as the elastic modulus varies within the interval of 400–2000 kPa (Figure 7C). The graph shows that as the elastic modulus decreases, the reduction trend in plantar peak stress slows down. When the elastic modulus decreases to approximately 800 kPa, the plantar peak stress begins to reach a plateau, approaching a horizontal asymptote, with a predicted peak stress value of 11.50 kPa, reflecting a 74.68% decrease compared to barefoot conditions.

### FEA in ML shear direction

**FIGURE 8:**
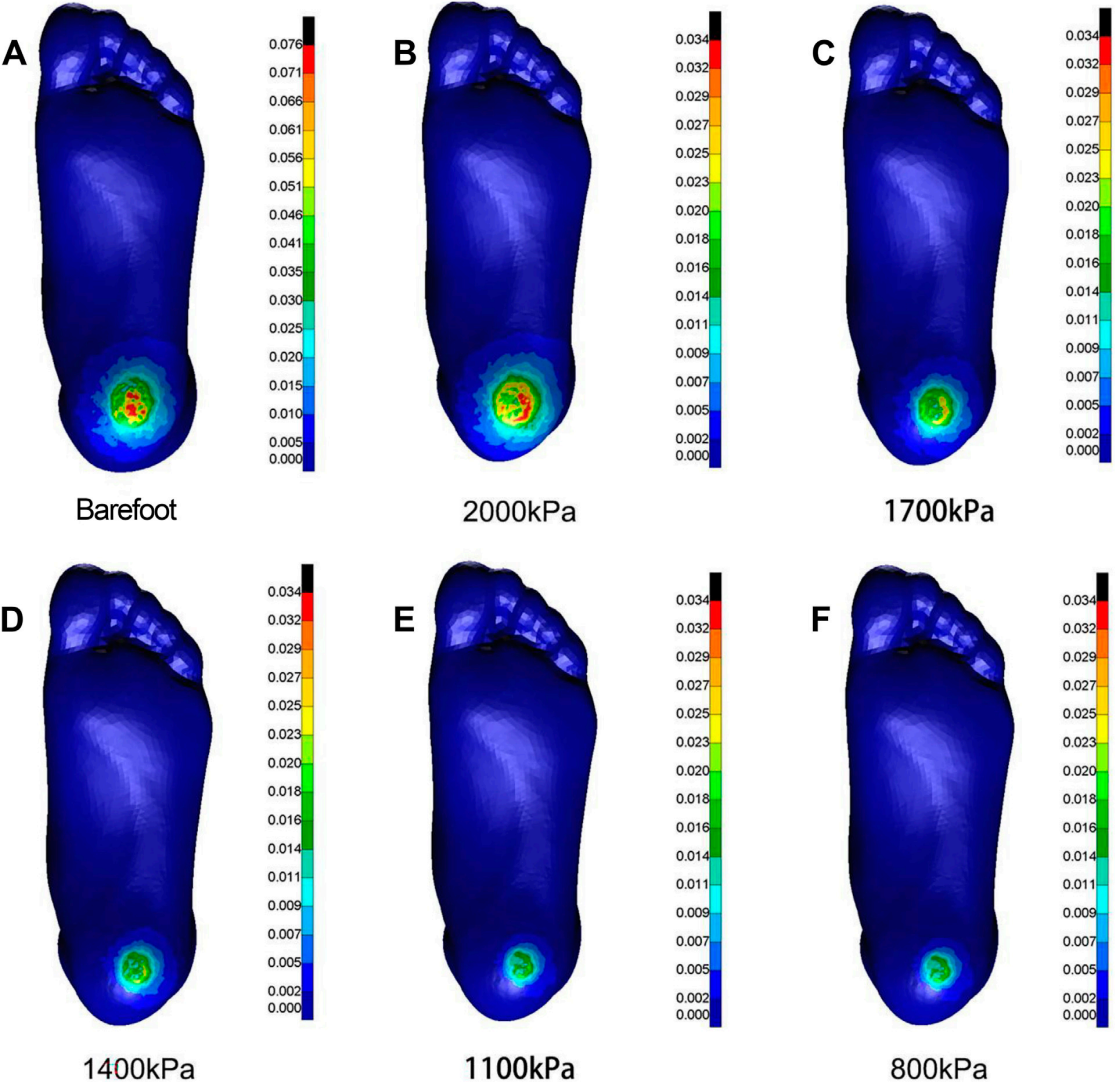
Cloud map of Mises stress under mediolateral shear towards different heel cushion elastic moduli (**B**: 2000kPa; **C**: 1700kPa; **D**: 1400kPa; **E**: 1100kPa; **F**: 800kPa) compared to barefoot state **(A)**. The color bar unit is MPa.

**FIGURE 9:**
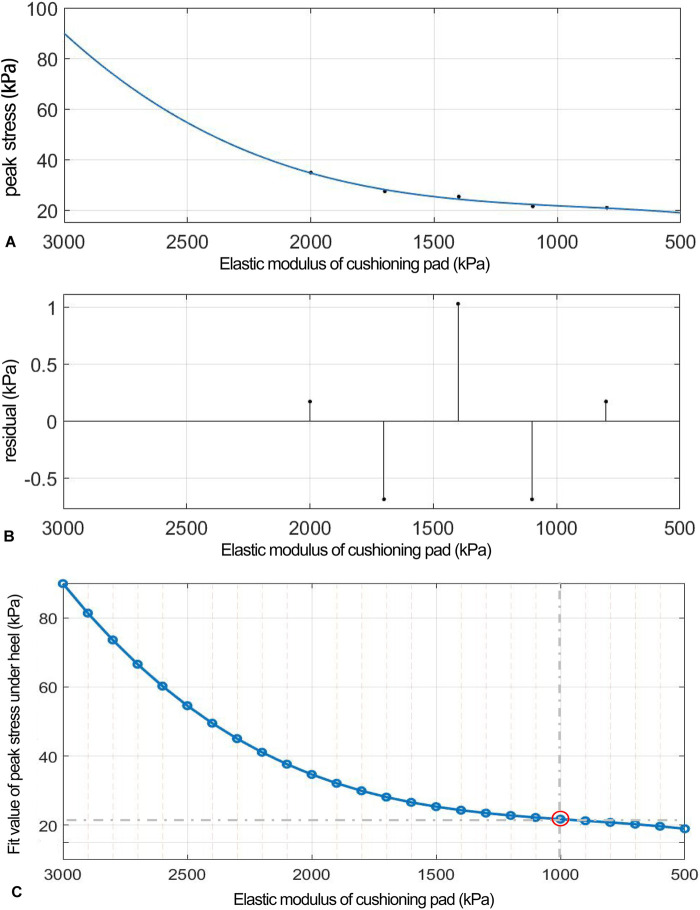
**(A)** Polynomial fitting curve between peak shear stress and shear elastic modulus of the heel cushion pad. **(B)** Residual plot. **(C)** Using the fit equation, the change curve of predicted peak shear stress on the plantar surface with changes in the mediolateral shear elastic modulus of the heel cushion pad within the range of 500–3,000 kPa. When the elastic modulus decreases to approximately 800 kPa, the plantar peak shear stress gradually reaches a plateau. ML, mediolateral.

As the elastic modulus of the heel cushion pad decreased, the distribution of ML shear stress in the stress cloud image also reduced (see Figure 8). Compared to barefoot conditions, the heel cushion pad with an ML shear elastic modulus of 800–2000 kPa demonstrated significant buffering effects against ML shear forces, with a notable reduction in peak shear stress (see Supplementary Figure S1C; Table 4, decreasing from 76.03 kPa to a range of 20.97–34.85 kPa, representing a decrease of 54.16%–72.42%). Furthermore, as the elastic modulus of the heel cushion pad uniformly decreased, the rate of decline in plantar peak stress gradually slowed, indicating that the relationship between the two is not simply linear. Therefore, a polynomial fitting was performed to illustrate the relationship between the two, as shown in Figure 9A, resulting in the following regression equation:

fx=p1*x^3+p2*x^2+p3*x+p4.

**TABLE 4:** Relationship between the peak AP shear stress and elastic moduli of the heel cushion pad.

Elastic modulus (kPa)	Peak shear stress (kPa)	Percentage decline (compared to barefoot)
Barefoot	76.03	0.00%
2,000	34.85	54.16%
1,700	27.44	63.91%
1,400	25.36	66.64%
1,100	21.55	71.66%
800	20.97	72.42%

Footnote: ML, mediolateral.

(“x” is standardized using a mean of 1,400 and a standard deviation of 486.3. The regression coefficients and their respective 95% CIs are as follows: p1 = 0.692 [−18.32, 19.71], p2 = 2.13 [−10.07, 14.33], p3 = 4.38 [−23.04, 31.8], and p4 = 24.33 [11.61, 37.05]).

The fitted regression line closely matches the original data points, as indicated by the small residuals in the residual plot (Figure 9B), suggesting excellent model fitting. The adjusted R2 value is 0.9344, and the RMSE is 1.437, further confirming the excellent goodness of fit. Using the above regression equation, a curve graph is drawn to predict the plantar ML peak shear stress as the elastic modulus varies within the interval of 500–3,000 kPa (Figure 9C). The graph shows that as the elastic modulus decreases, the reduction trend in plantar peak stress slows down. When the elastic modulus decreases to approximately 1,000 kPa, the plantar peak stress begins to reach a plateau, approaching a horizontal asymptote, with a predicted peak shear stress value of 21.74 kPa, reflecting a 71.41% decrease compared to barefoot condition.

## Discussion

This study established FE models of the heel region and heel cushion pad based on the stress–strain material constitutive relationships of the heel soft tissue measured in three dimensions in previous research. The simulation involved setting gradient elastic moduli for the heel cushion pad in each dimension, resulting in curves showing the changes in surface peak stress with varying elastic moduli of the heel pad. The trends observed in these curves indicate that within the preset range of heel cushion pad elastic moduli, peak compressive, AP shear, and ML shear stresses in the heel area can be effectively reduced. This study fitted polynomial regression curves and found that in the three dimensions, as the elastic modulus of the heel cushion pad decreases, the plantar peak stress tends to stabilize and reach a relative plateau, suggesting that further reduction in elastic modulus does not provide more stress-buffering performance in the corresponding dimensions.

The stiffness of insoles and their pressure-buffering effects have always been a hot topic of interest. Initially, designs for pressure-relieving insoles for the sole were mostly made of uniform material properties with flat surfaces (Frick and Rochman, 2004; Gibson et al., 2014). However, researchers gradually discovered that this did not provide optimal pressure relief during actual use as the uneven distribution of stress on the sole due to the unique anatomical structure of the foot led to relatively high-pressure areas, such as the forefoot and heel regions. Therefore, each area of the sole should be designed with different material properties and structural components according to its specific stress distribution characteristics. In recent years, some studies have focused on developing insoles with varying stiffness that adapt to the stress distribution characteristics of the sole or adding auxiliary structures (such as metatarsal pads, heel pads, arch supports, and medial/lateral wedges) in high- or low-pressure areas to improve pressure-buffering effects (Collings et al., 2020; Chen et al., 2015; Guldemond et al., 2007). Although adding extra insole components has been proven effective in improving overall pressure relief in many studies, the discontinuous change in stiffness inside the insole can affect the comfort of the insole when in contact with the foot during actual wear. Therefore, some researchers are also trying to design cushioning insoles with a continuous gradient of stiffness (Qiu et al., 2011). Jafarzadeh et al. (2021) used the FE method to iteratively design the stiffness and surface shape of each part of the insole to optimize pressure relief. The final insole design had varying stiffness in each part, and the surface topography matched the sole optimally; after iterative calculations for stiffness and shape, the insole model showed significant changes in the plantar stress distribution, which was more evenly distributed across the entire sole, and peak stress was significantly reduced.

With the mature technology and high academic interest in plantar compressive cushioning insoles for diabetic patients, a wealth of related research and products is available. In contrast, attention to plantar shear stress has only recently increased, with limited prior research and complex technology, making related studies and available products rare. A systematic search and screening of various databases yielded only five English-language articles (Lavery et al., 2012; Lavery et al., 2005; Lavery et al., 2015; Belmont et al., 2013; Wrobel et al., 2014) on shear force-relieving insoles, with only two types of products mentioned [GlideSoft (Lavery et al., 2012; Lavery et al., 2005; Lavery et al., 2015) and dynamic foot orthosis (DFO) (Belmont et al., 2013; Wrobel et al., 2014)]. Lavery et al. (2005) designed the GlideSoft insole in 2005, consisting of three layers, namely, a top 1.5-mm PLZ material layer, a bottom 3.2-mm Plastazota^®^ foam layer, and a middle layer made of two thin sheets of fiberglass coated with Teflon^®^, providing low friction for lateral movement during loading. The authors confirmed the shear-relieving performance of GlideSoft, reducing peak shear force by more than 50% compared to standard insoles. Lavery et al. (2012) studied 299 diabetic neuropathy patients, assigning them to either GlideSoft (n = 149) or standard insoles (n = 150), and found a reduced risk of ulcers with GlideSoft, with a hazard ratio of 3.47. In 2015, another study by the same authors on DFU patients using therapeutic sandals (n = 23), TCC (n = 23), and GlideSoft insole (n = 27) found that GlideSoft had poor healing outcomes and patient satisfaction. The DFO insole, designed by Belmont et al. (2013), aims to reduce forefoot shear force, torque at metatarsal heads, and plantar pressure. It was composed of three layers, namely, a nylon mesh base, a mid-layer of pressure-absorbing materials such as Plastazote/PPT/silicone, and an upper layer of Darlexx fabric, with complete separation between the mid and bottom layers in the forefoot region. Clinical trials have shown that DFO reduces shear force in the hallux and metatarsal heads by more than 200% compared to standard insole. Wrobel et al. (2014) measured skin temperature at different foot regions after walking 200 m with both types of insoles and found that DFO significantly reduced temperature in the forefoot and midfoot compared to the standard insole.

Our preliminary research revealed that plantar soft tissue is not uniformly isotropic in terms of vertical compression, AP shear, and ML shear. The shear modulus is significantly higher than the vertical compressive modulus. Previous insole designs have relied on isotropic materials that cannot provide optimal shear force relief. Hence, this study used FE methods to preset a gradient series of elastic moduli in three dimensions for cushion materials and observed the changes in peak pressure and shear stresses at the heel surface. Curve equations were fitted between heel cushion pad moduli and peak stresses to guide and judge the selection of cushion materials in three dimensions. The regression curves indicated that when the elastic modulus of the heel cushion pad decreased to 400 kPa in the vertical direction, 800 kPa in the AP shear direction, and 1,000 kPa in the ML shear direction, the stress-buffering effects reached a plateau, achieving 66.59%, 74.68%, and 71.41% of the stress-buffering effect, respectively, compared to barefoot status. This suggests that selecting the above elastic modulus values for the three dimensions could achieve better buffering, and further reducing the elastic modulus does not offer much additional benefit to the buffering performance.

This study used biomechanical principles and structural engineering concepts to conduct detailed three-dimensional simulation of the biomechanical characteristics of heel cushion pads with different elastic moduli, deriving meaningful biomechanical conclusions. The involvement of the FE method reduced the need for a large number of cadaver or animal experiments, allowing for theoretical research into the changes in biomechanical behavior. This helps advance the development of heel cushions for people with diabetes. However, as a theoretical research method, the FE method comes with certain assumptions and hypotheses. To ensure a rigorous scientific attitude, designing a heel cushion pad with superior material properties and excellent performance still requires experimental validation and clinical trials to optimize and achieve an ideal state of research. Additionally, the current static simulation approach may not fully capture dynamic gait variations (e.g., transient impacts during walking). Thus, to model time-dependent gait events (e.g., heel strike to toe-off phases), we plan to incorporate transient dynamics analysis validated with experimental motion capture data. Finally, there is a reliance on a specific set of loading conditions (quasi-static forces; vertical pressure, AP shear, and ML shear), which may not represent the full spectrum of real-world diabetic foot scenarios. So, we need to collaboratively test the model against larger-scale *in vivo* patient data from diabetic populations to generalize findings, including longitudinal studies to assess long-term biomechanical effects.

## Conclusion

This study, based on the anisotropic constitutive relationship of plantar soft tissue measured in previous experiments, initially explored the elastic moduli of heel cushion pads in three dimensions using FEA, aiming to achieve synchronous pressure and shear force cushioning effectiveness. The results showed that the plantar peak stress decreases with the decrease in the elastic modulus of the heel cushion pad, reaching a relative plateau where further decrease provides minimal additional buffer effect gain. This suggests that the inflection point of the elastic modulus before reaching the plateau may be the optimal choice for industrial conversion of the heel cushion pad.

## Data Availability

The raw data supporting the conclusions of this article will be made available by the authors, without undue reservation.
