# Supplementary material for: Finite-element analysis-based design and efficacy assessment of a three-dimensional anisotropic heel cushioning pad for diabetic foot management
Source: Front Bioeng Biotechnol. 2025 Nov 19;13:1694935. doi: 10.3389/fbioe.2025.1694935 (PMC12673281; doi:10.3389/fbioe.2025.1694935)
Supplement: Supplementary file 1 [file Table1.docx]

| **Supplementary Table S1.** Material parameters of ligaments and tendons | | | |
| --- | --- | --- | --- |
| **Ligaments /tendons** | **Elastic modulus（MPa）** | **Poisson's ratio** | **Stiffness（N/mm）** |
| anterior tibiofibular ligament | 260 | 0.4 | 90.78 |
| posterior tibiofibular ligament | 260 | 0.4 | 82 |
| anterior talofibular ligament | 255.5 | 0.4 | 141.8 |
| posterior talofibular ligament | 216.5 | 0.4 | 82 |
| calcaneofibular ligament | 512 | 0.4 | 63 |
| anterior talotibial ligament | 184.5 | 0.4 | 122.6 |
| posterior talotibial ligament | 99.5 | 0.4 | 60 |
| tibiocalcaneal ligament | 512 | 0.4 | 63 |
| tibionavicular ligament | 320.7 | 0.4 | 39.1 |
| other ligaments /tendons | 260 | 0.4 | 70~90 |
