# Supplementary figures and images for: Finite-element analysis-based design and efficacy assessment of a three-dimensional anisotropic heel cushioning pad for diabetic foot management
Source: Front Bioeng Biotechnol. 2025 Nov 19;13:1694935. doi: 10.3389/fbioe.2025.1694935 (PMC12673281; doi:10.3389/fbioe.2025.1694935)

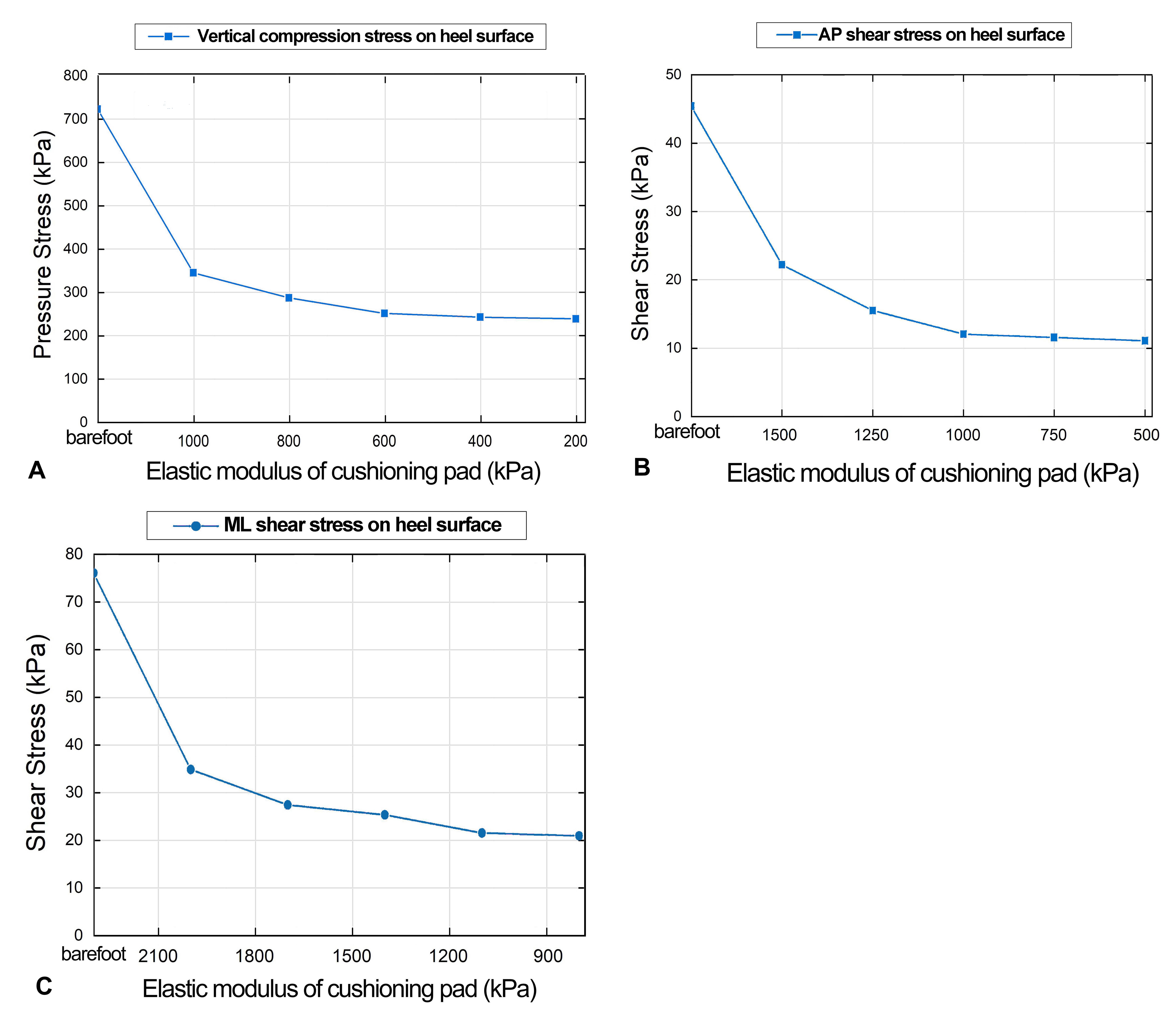

Supplement: Supplementary file 2 [file Image1.jpeg]
